# Outlines of Rural Hygiene

**Published:** 1898-02

**Authors:** 


### BOOK NOTICES.

OUTLINES OF RURAL HYGIENE. For Physicians, Students, and Sanitarians. By Harvey B. Bashore, M. D., Inspector for the State Board of Health of Pennsylvania. With an Appendix on "The Normal Distribution of Chlorine," by Professor Herbert E. Smith, of Yale University. Illustrated with twenty (20) engravings. 5½x8 inches. Pages vi-84. Extra cloth, 75 cents net. The F. A. Davis Co., Publishers, 1914-16 Cherry Street, Philadelphia; 117 West Forty-second Street, New York city; 9 Lakeside Building, 218-220 South Clark Street, Chicago, Ill.

This admirable little book deals with the question of water-supply in the country from wells, rivers, and springs. The contamination with sewage and other deleterious matters is clearly shown, and methods of improvement in the quality of the water suggested. The tilting of strata and the result in contaminating underground streams of water by drainage into them of organic matter from the surface is clearly shown. The question of the disposal of excreta, slop-waters, garbage, ashes, and other refuse is discussed and a method of disposing of it suggested. The chapter on "soils" is very condensed and very interesting, and summarizes the new views of that subject. We cannot refrain from noting a few of these teachings. The uppermost layer of soil is called "surface soil." It is largely made up of *humus*, which is the product of decomposition of animals and plants.

This surface soil is full of life—bugs, worms, and bacteria.

This "surface soil" is a vast laboratory for processes that feed and clothe the race.

The "surface soil" is infested with bacteria which are the great scavengers, and bring about decay and disorganization which is called *nitrification*, and the products are ammonia, nitrites, and nitrates, the last being food for plants. Sewage farms, filter beds, and earth closets are processes of *nitrification*.

Excessive heat destroys the nitrifying bacteria, and therefore oven-dried earth is of no use as a disinfectant. It can act only as an absorbent.

Germicides destroy nitrifying bacteria. Hence carbolic acid added to a manure heap defeats the object sought, and prevents the nitrification of the organic matter and its consequent neutralization as an element dangerous to health.

The foregoing comments do not exhaust the material of the book, but there is not space or time for more. So many of the subscribers to this journal are country practitioners, to whom the subjects treated in this book are of the hightest importance, that this notice has been so written as to enable them to see for themselves how valuable it must be to them, every one.

